# Supplementary material for: Functional muscle networks as biomarkers of post-stroke motor impairment and therapeutic responsiveness
Source: eLife. 2026 Jul 23;14:RP108509. doi: 10.7554/eLife.108509 (PMC13395455; doi:10.7554/eLife.108509)
Supplement: Peer review history

# Editor report: eLife Assessment

**DOI:** 10.7554/eLife.108509.4.sa0

**Authors:** Leonardo Elias

**Affiliations:** Universidade Estadual de Campinas (UNICAMP) Brazil

**Keywords:** Important, Solid

This **important** work employed a recent functional muscle network analysis to evaluate rehabilitation outcomes in post-stroke patients. The research direction is relevant and supported by **solid** evidence from gross motor function assessment. The framework is a step toward standardized assessment of motor recovery in the rehabilitation process, but future studies would focus on linking functional recovery to muscle interaction biomarkers to provide more physiologically grounded interpretations.

# Referee report: Reviewer #1 (Public review)

**DOI:** 10.7554/eLife.108509.4.sa1

This study addresses an important clinical challenge by proposing muscle network analysis as a tool to evaluate rehabilitation outcomes. The research direction is relevant and the findings suggest further research.

The revised manuscript included additional methodological details and a supplementary comparison with conventional NMF.

Comments on latest version:

No additional comments.

# Referee report: Reviewer #2 (Public review)

**DOI:** 10.7554/eLife.108509.4.sa2

This study presents an important analysis of how interactions between muscles can serve as biomarkers to quantify therapeutic responses in post-stroke patients. To do so, the authors employ an information-theoretical metric (co-information) to define muscle networks and perform cluster analysis.

Comments on revised version.

Thank the authors for the carefully revised article. I have no further comments.

# Author comment: Author response

**DOI:** 10.7554/eLife.108509.4.sa3

**Authors:** David O'Reilly, Giorgia Pregnolato, Andrea Turolla, Pawel Kiper, Ioannis Delis, Giacomo Severini

**Affiliations:** University of Leeds Leeds United Kingdom; University College Dublin Dublin Ireland; University of Bologna Bologna Italy; Healthcare Innovation Technology Lab, IRCCS San Camillo Hospital Venice Italy; National Technical University of Athens Athens Greece; University College Dublin Dublin Ireland

The following is the authors’ response to the previous reviews

**Public Reviews:**

**Reviewer #1 (Public review):**

While the revised manuscript includes additional methodological details and a supplementary comparison with conventional NMF, it would be great if the authors could add the point below as limitations in the manuscript or change the title and abstract accordingly, since core issues remain:

(1) The study claims to evaluate rehabilitation outcomes without demonstrating that patients actually improved functionally

(2) The comparison with existing methods lacks the quantitative rigor needed to establish superiority

(3) The added value of this complex framework over much simpler alternatives has not been demonstrated

The strength of evidence supporting the main claims remains incomplete. I would encourage the authors to consider discussing these points

(1) including or adding a limitation section about functional outcome measures that go beyond clinical scale scores, (2) providing/discussing quantitative benchmarks showing their method outperforms alternatives on specific, predefined metrics, and (3) clarifying the clinical pathway by which these biomarkers would inform treatment decisions.

We thank the reviewer for their thoughtful consideration of our study, and now better understand their perspective on the limitations of the study. We now see the importance of the aspects of functional recovery the reviewer has highlighted in the context of our work, as the clinical measure we focused on (i.e. FMA-UE) does not capture recovery at activities and participation-levels of the ICF model. Although the FMA-UE is a gold standard measure for assessing post-stroke recovery, it is limited in scope to gross motor functions.

To more accurately describe the aspects of functional recovery the biomarkers in our study reflected, we have extensively revised the terminology used throughout the paper. For example, in the abstract we now include “…From these patterns, we derived new biomarkers that stratified patients by gross motor impairment severity and therapeutic responsiveness, each associated with unique physiological signatures.” and go on in the abstract to now highlight the limited scope of the evidence towards functional recovery more broadly also: “Future research should employ this framework to identify biomarkers of activities- and participation-related functional recovery.” In the rest of this paper, we also make this distinction clear, for example at the beginning of the results section: “The cohort of stroke survivors overall experienced a statistically significant increase at FMA-UE (Pre-treatment: 43.1±13.2, Post-treatment: 49.1±13.6 (t = -7.84, *p<0.001*)), representing a clinically important effect from rehabilitation on the gross motor functions of the upper-extremity (Page et al., 2012).” Finally, we have now added a limitations section, as the reviewer advised, where we specifically detail the scope of evidence provided in this study and how future research could build on it:

“Limitations

Although the FMA-UE is a gold standard measure of post-stroke treatment outcomes (Meyer et al., 1975; Page et al., 2012), it does not capture the impact of rehabilitation on patients' ability to perform activities-of-daily-living or to participate in daily life. Hence, interpretations of the identified biomarkers are currently limited to gross motor function impairment and recovery. Future research should employ this framework to quantify biomarkers that correspond to other important aspects of patients' recovery (e.g. functional independence, subjective experiences), thus offering a more complete evidence base for its clinical utility.”

With these changes, we believe this manuscript more accurately describes the scope of the biomarkers analysed and hence no longer offers incomplete evidence towards stated claims.

Regarding the reviewers second and third points concerning the validity and advantages of this framework against current approaches, in this study we applied a framework that builds on two previous papers (O’Reilly & Delis, 2022; O’Reilly & Delis, 2024). In both of these papers, we compared basic aspects of the framework to the current prevailing approach and most relevant comparative for this line of research in muscle synergy analysis, that is non-negative matrix factorisation (NNMF).

To briefly outline this existing foundation of evidence, in O’Reilly & Delis, 2022 we dedicated most of the discussion section (i.e. sections 4.1 and 4.2) along with a supplementary materials document to comparisons with this approach. In section 4.1, we illustrate the continuity of this framework with what has come before in simpler methodologies such as NNMF and then went on in section 4.2 to show the novel insights and opportunities that can generated from our framework. Additionally, in the corresponding supplementary materials of that paper, we directly compared our framework with three different models from the established NNMF approach (i.e. spatial, temporal and space-time) by applying them to the same datasets, again highlighting points of congruence and additional utility with our framework. Building on this work, in O’Reilly & Delis, 2024, we also ensured that developments of this framework both align with previous research and credibly improve upon them methodologically. For example, Fig.5 and Fig.6 and associated text of that paper illustrates a direct comparison of our framework with the NNMF methodology, showing that it provides additional functional and physiological relevance and predictive capacity to the components extracted. Further, in the results of that paper we also directly compared the generalisability of the extracted components when extracted using our chosen dimensionality reduction approach vs other approaches promoted in the neurosciences more generally (e.g. non-negative Canonical-Polyadic (CP) tensor decomposition (Williams et al (2018))), showing that we extracted more robust components across participants and tasks.

This previous work directly supports the credibility of basic aspects of the framework and its outputs compared to other established approaches. We have directed readers towards this previous research in the methods section of the current study: “Further comparisons with conventional approaches can be found in our previous work developing this framework (O’Reilly & Delis, 2022; O’Reilly & Delis, 2024).”

Continuing, and building on the credibility of these basic aspects of the framework, as the reviewer previously suggested, we have included additional supplementary material in the current study illustrating how the biomarkers generated from our approach could not be found using conventional methods. In these supplementary analyses, we employed a much simpler but conceptually aligned pipeline involving NNMF and agglomerative clustering on the same dataset and directly compared the outputs, highlighting commonalities and where our approach improves significantly upon this established approach. The advancements we demonstrate here also address recognised limitations in the current NNMF approach for clustering activation coefficients (see Scano et al 2017), a point we now highlight directly in the revised manuscript:

“Enhanced interpretability of extracted components and clusters.

As our framework maps muscle interactions to a specific task parameter, we yield population-level motor components that correspond more consistently to meaningful biomechanical and physiological functions that can be interpreted across the dimensions of the specified task parameter. The proposed clustering approach also offers enhanced interpretability, addressing key limitations in the application of clustering approaches to the activation space of conventional muscle synergy analysis (e.g. different activation timings) (Scano et al., 2017).”

Taken together, we believe the extensive comparisons made in our previous work on this framework and direct comparisons made in this study provide sufficient evidence towards its added value for the field beyond current approaches.

References

Ó’Reilly D, Delis I. A network information theoretic framework to characterise muscle synergies in space and time. Journal of Neural Engineering. 2022 Feb 1;19(1):016031.

O'Reilly D, Delis I. Dissecting muscle synergies in the task space. Elife. 2024 Feb 26;12:RP87651.

Williams et al. (2018) Unsupervised discovery of demixed, low-dimensional neural dynamics across multiple timescales through tensor component analysis. Neuron 98:1099–1115.

There are specific, relatively minor points, that require attention

The authors write: "we did not focus on such complementary evidence in this study." This is a weakness for a paper claiming to provide "biomarkers of therapeutic responsiveness." The FMA-UE threshold defines responders, but there's no independent validation that patients actually functioned better in daily life. Can you please clarify?

See above for our response on this important aspect of the reviewer’s commentary.

Maybe I missed the exact point about this, but with the added NMF plot, the authors list 'lower dimensionality' among their framework's advantages, but the basis for this claim is not clear because given that 12 network components were extracted compared to 11 "conventional" synergies. Can you please clarify, as it is not clear. You claim 'lower dimensionality' as an advantage of the proposed framework (in the Supplementary Materials), yet you extracted 12 components (5 redundant + 7 synergistic networks) compared to 11 synergies from the conventional NMF approach, which does not support a clinical / outcome advantage of this method. Please clarify.

We agree with the reviewer that this statement is confusing given that overall, across separate decompositions for redundant and synergistic networks compared to the single decomposition using NNMF, there are more dimensions to consider in our frameworks output. For this reason, we have removed this statement from the updated manuscript.

**Reviewer #2 (Public review):**

This study presents an important analysis of how interactions between muscles can serve as biomarkers to quantify therapeutic responses in post-stroke patients. To do so, the authors employ an information-theoretical metric (co-information) to define muscle networks and perform cluster analysis.

I thank the authors for improving the clarity of the Methods section; the newly added Figure 5 is very helpful.

One minor suggestion is that the authors should avoid overloading the notation "m" for both the EEG measurement and the matrix of II values (Eq. 1.1), which I now realise was the source of some of my initial confusion. I suggest that the authors use separate notation for these two quantities.

We thank the reviewer for their consideration and positive outlook on our study. In the updated manuscript, we have adjusted the notation for equation 1.1 so that it doesn’t cause confusion with earlier text.

**Recommendations for the authors:**

Reviewer #1 raised critical concerns about the method's ability to identify functional improvements resulting from rehabilitation protocols. In this regard, the study's translational impact remains limited, and the authors should address these limitations in a revised version. The Reviewing Editor and both reviewers agree that the "Strength of Evidence" of the manuscript cannot be improved without a major revision, given the above-mentioned aspects.
